# Supplementary material for: The genome sequence of the Silver-barred Sober moth, Aproaerema taeniolella (Zeller, 1839)
Source: Wellcome Open Res. 2024 Sep 3;9:500. [Version 1] doi: 10.12688/wellcomeopenres.22890.1 (PMC11576566; doi:10.12688/wellcomeopenres.22890.1)
Supplement: Peer review history

# Reviewer report: Reviewer response for version 1

**DOI:** 10.21956/wellcomeopenres.25206.r109094

**Published:** 2024-11-19

**Authors:** David E.K. Ferrier

**Affiliations:** 1 School of Biology, University of St Andrews, St Andrews, Scotland, UK

The rationale for sequencing this genome as part of the Darwin Tree of Life initiative is clear. This moth is one of the many that is being sequenced as part of the dense sampling from the efforts of the Wytham Woods DToL moth team, and this additional species is part of an unprecedented dataset of moth genomic data suited to analyses that impact multiple biological research areas.

 The photograph of the specimen is not very clear, and would have benefited from being a higher magnification. Perhaps this comment can be taken onboard for future collections and documentation of samples since it presumably cannot be remedied for this report?

 The protocols and Materials and Methods are briefly described and rely heavily on links to other sources of information. This is a fairly standard approach in these Wellcome Open Research reports and thus meets the standard required.

 The datasets are all of good quality and are accessible via the links provided.

 Three typos are present:

 1) “taeniolella” is spelt incorrectly at the start of the second Background paragraph.

 2) On the fifth line of the Background second paragraph ‘sometime’ should be ‘sometimes’.

 3) The final sentence in the section ‘Genome annotation report’ should be, “There are an average of 1.01….”.

Are sufficient details of methods and materials provided to allow replication by others?

Yes

Is the rationale for creating the dataset(s) clearly described?

Yes

Are the datasets clearly presented in a useable and accessible format?

Yes

Are the protocols appropriate and is the work technically sound?

Yes

Reviewer Expertise:

NA

I confirm that I have read this submission and believe that I have an appropriate level of expertise to confirm that it is of an acceptable scientific standard.

# Reviewer report: Reviewer response for version 1

**DOI:** 10.21956/wellcomeopenres.25206.r105730

**Published:** 2024-11-04

**Authors:** Dejian Zhao

**Affiliations:** 1 Yale School of Medicine, New Haven, CT, USA

The authors utilized advanced sequencing methods, including PacBio HiFi long-read sequencing, Illumina sequencing, and RNA sequencing, and presented a chromosome-level genome assembly of the Silver-barred Sober moth, *Aproaerema taeniolella* (Zeller, 1839), laying an essential foundation for genomic, evolutionary, and functional research. As part of the Darwin Tree of Life Project, it will deepen our understanding of the diversity of life. Below are a few points that require further clarification.

 (1). How many chromosomes does this species have? Are there any cytological data, such as karyotypes, or previous references about the chromosome number? If so, please either cite the reference or present the experimental result.

 (2). Did the authors do any experiment such as flow cytometry to estimate the genome size of this species? Does the genome assembly size align with the experimental result?

 (3). The 2nd paragraph of the Background section starts with " *Aproaerema taeniolla *is a blackish moth". It seems the species name was misspelled. taeniolla should be taeniolella?

 (4) Were all specimens (ilAprTaen1, ilAprTaen2, and ilAprTaen3) female? Please clarify in the section of Sample acquisition.

 (4). In the section of Assembly curation, "The assembly was decontaminated using the Assembly Screen for Cobionts and Contaminants (ASCC) pipeline (article in preparation)." The authors should provide some more details about how it was decontaminated.

 (5). In the section of Assembly curation, "The sex chromosome was identified by read coverage statistics. The process is documented at https://gitlab.com/wtsi-grit/rapid-curation (article in preparation)." The authors should briefly describe how it worked rather than simply post a link here.

 (6) Based on Table 3 (Table 3. Chromosomal pseudomolecules in the genome assembly of *Aproaerema taeniolella*, ilAprTaen1.), the sex chromosome Z is the largest (39.05 Mb). Is it a common phenomenon for moth? Are there any other species showing that the sex chromosome is the largest? Are there any known sex-determining genes on the Z chromosome? Can the authors add one more column to the Table 3 showing the number of genes on each chromosomal pseudomolecules?

Are sufficient details of methods and materials provided to allow replication by others?

Partly

Is the rationale for creating the dataset(s) clearly described?

Yes

Are the datasets clearly presented in a useable and accessible format?

Yes

Are the protocols appropriate and is the work technically sound?

Yes

Reviewer Expertise:

genome assembly, functional genomics; next generation sequencing, PacBio sequencing

I confirm that I have read this submission and believe that I have an appropriate level of expertise to confirm that it is of an acceptable scientific standard, however I have significant reservations, as outlined above.

# Reviewer report: Reviewer response for version 1

**DOI:** 10.21956/wellcomeopenres.25206.r98837

**Published:** 2024-10-08

**Authors:** Yu-Feng Huang

**Affiliations:** 1 National Chung Hsing University, Taichung, Taiwan

This study presents a comprehensive and well-annotated genome sequence of *Aproaerema taeniolella*, a micro-moth species of ecological significance. The authors utilized state-of-the-art sequencing technologies, including Pacific Biosciences HiFi long-read sequencing and Illumina Hi-C short-read sequencing for contig scaffolding, to generate a high-quality, chromosomally complete genome assembly. Additionally, RNA sequencing using the Illumina platform was performed to assist with genome annotation. This dataset represents a valuable contribution to the Darwin Tree of Life Project, providing crucial genomic insights that will support future research in biodiversity, evolutionary biology, and conservation efforts. However, several key issues need to be addressed to improve the clarity and impact of the study.

 First, while the authors mention the use of PacBio long-read sequencing, Illumina Hi-C short-read sequencing, and Illumina RNA sequencing, the specific role of RNA sequencing in this genome project is not clearly illustrated. The manuscript should clarify whether RNA sequencing data was used to evaluate or improve genome annotation.

 Second, the paragraph lacks clarity on whether the circularity of the mitochondrial genome has been confirmed. This point should be explicitly addressed to ensure readers fully understand the assembly of the mitochondrial genome.

 Additionally, there is an inconsistency in the terminology used for tools assessing the assembly metrics. Both "MERQURY.FY" and "Merqury" are mentioned, but they appear to refer to the same tool. The authors should ensure consistent use of terminology throughout the manuscript.

 Furthermore, while the authors state that the sex chromosome was identified by read coverage statistics, this explanation lacks sufficient detail for readers. A more thorough description of the methods used to identify the sex chromosome would enhance understanding.

 In the "Evaluation of the final assembly" section, the organization of the paragraph requires revision. For instance, nf-core is an open-source platform for pipelines developed using the Nextflow framework, while Docker and Singularity are containerization solutions, and Conda and Bioconda provide virtual environments. Rather than simply listing the tools used, the authors should explain how these tools were implemented in a cohesive and practical workflow, making the approach more accessible to readers. This would provide greater insight into the feasibility and replicability of the methods employed in this project.

Are sufficient details of methods and materials provided to allow replication by others?

Yes

Is the rationale for creating the dataset(s) clearly described?

Yes

Are the datasets clearly presented in a useable and accessible format?

Yes

Are the protocols appropriate and is the work technically sound?

Yes

Reviewer Expertise:

I conduct research using next-generation sequencing in the areas of genome assembly, gene expression analysis, and cancer biology.

I confirm that I have read this submission and believe that I have an appropriate level of expertise to confirm that it is of an acceptable scientific standard, however I have significant reservations, as outlined above.
